# Qualitative Study of Nutritional Support-Related Perceptions and Preferences Among Persons Affected by TB, Family Caregivers, and Healthcare Providers in India

**DOI:** 10.3390/tropicalmed10040114

**Published:** 2025-04-21

**Authors:** Balaji Ramraj, Karikalan Nagarajan, Debjani Ram Purakayastha, Major Madhukar, Makesh Kumar, Neha Raj, Sarath Kumar, Banappa S. Unger, Nithin Rajamani, Sampada Dipak Bangar, Murugesan Periyasamy, Hansraj Choudhary, Yasaswany Santhoshkumar, Ramesh Kumar, Seema Sahay, Nivedita Gupta, Chandrasekaran Padmapriyadarsini

**Affiliations:** 1 ICMR National Institute for Research in Tuberculosis, Chennai 600031, India; balaji.ramraj@icmr.gov.in (B.R.); makesh.kumar@icmr.gov.in (M.K.); karmansri123@gmail.com (S.K.); periyasamy.m@icmr.gov.in (M.P.); yasaswany.s@icmr.gov.in (Y.S.); santhana.k@icmr.gov.in (R.K.); pcorchids@gmail.com (C.P.); 2 Academy of Scientific and Innovative Research (AcSIR), Ghaziabad 201002, India; banappau@icmr.gov.in (B.S.U.); sdhayarkar@nariindia.org (S.D.B.); ssahay@nariindia.org (S.S.); 3 Indian Council of Medical Research, New Delhi 110029, India; rp.debjani@gmail.com (D.R.P.); hansraj.icmr@gmail.com (H.C.); guptanivedita.hq@icmr.gov.in (N.G.); 4 ICMR Rajendra Memorial Research Institute of Medical Sciences, Patna 800007, India; madhukarcaptpatna@gmail.com (M.M.); neharaj190196@gmail.com (N.R.); 5 ICMR National Institute of Traditional Medicine, Belagavi 590010, India; 6 ICMR National Institute of Immunohematology, Mumbai 400012, India; nithinrajamani.icmr@gmail.com; 7 ICMR National Institute of Translation Virology and AIDS Research (Formerly ICMR-NARI), Pune 411026, India

**Keywords:** nutrition, tuberculosis, family caregivers, healthcare providers, India, DBT

## Abstract

Evidence on the implementation aspects of nutritional support interventions for persons with TB in India is limited. This qualitative study employed focus group discussions with persons with TB (n = 71), their family caregivers (n = 17), and healthcare providers (n = 18). The study was conducted from August 2023 to April 2024 in five states in India. Participants’ knowledge, perceptions, and practices about nutritional intake, experiences, and expectations when accessing nutritional support were explored. Four nutrition-related themes emerged: (a) the experiences and perceptions of persons with tuberculosis and their caregivers, explained by their understanding of the importance of adequate nutrition and TB cures; (b) changes in food practices, explained by protein food adoption, alongside food insecurity experienced by those in poverty; (c) Direct Benefit Transfer (DBT)-related issues, explained by insufficiency and access-related gaps; and (d) preferred choices for nutrition support delivery, explained by less preference towards the involvement of intermediaries and a public distribution system alongside preference for the provision of nutrition through treatment facilities. Our findings underscore the importance of the provision of protein-rich food and an increase in financial support based on needs assessments. Mitigating the linkage and access gaps in DBT is needed. The delivery of ready-to-consume food through tuberculosis treatment facilities could be prioritized.

## 1. Introduction

Undernutrition is a risk factor that exacerbates the incidence and transmission of tuberculosis [1,2]. It continues to be a significant co-morbidity among individuals with tuberculosis in India. Individuals with tuberculosis who experience undernutrition and possess a low body mass index (BMI) are at a heightened risk for TB disease severity, drug toxicity, malabsorption, disease relapse, and mortality [2]. Further, household food insecurity contributes to an increased incidence of tuberculosis among household contacts and close social contacts [3]. Undernutrition is a critical factor that can lead to poor adherence to tuberculosis (TB) treatment, increasing the risk of developing drug-resistant TB. Inadequate nutrition weakens the immune system, making it more challenging for patients to withstand prolonged and intensive TB treatment regimens. Poor nutritional status can result in fatigue, reduced tolerance to medication side effects, and overall diminished well-being, leading to missed doses or the discontinuation of treatment. Comprehensive care for multidrug-resistant TB (MDR-TB) should include nutritional support to enhance treatment adherence and outcomes, particularly in vulnerable populations such as young children [4,5].

Recognizing the significance of adequate nutrition for eliminating tuberculosis, the Ministry of Health and Family Welfare of the Government of India implemented the Direct Benefit Transfer (DBT) program under its Nikshay Poshan Yojana scheme, which allocates monthly monetary support to individuals receiving treatment through the National TB Elimination Program (NTEP) to fulfill their nutritional needs [6].

To address the unfulfilled nutritional requirements of individuals with TB, the Pradhan Mantri TB Mukt Bharat Abhiyan (PMTBMBA) has introduced the Nikshay Mitra initiative [7]. Under this initiative, individuals, local bodies, and organizations are encouraged to adopt persons with TB and provide them with nutritional support. In addition, there have been independent nutritional support interventions which have shown positive impacts [8,9].

Many studies, including clinical trials, have demonstrated the positive effects of nutritional interventions for tuberculosis. Experiences in other high-TB-burden countries (Brazil, Russia, and Tanzania) have shown that the provision of food baskets (e.g., micronutrient or macronutrient food and energy supplements), nutritional advice, counseling, and incentives for buying food improved treatment adherence [10]. Studies conducted in South American countries have shown that financial incentives for nutrition had result in higher TB cure rates [11,12,13,14].

But evidence on the implementation-related gaps and challenges of nutritional interventions are scarce India [15]. Although multiple interventions are being implemented at a programmatic level to address the nutritional needs of persons with TB in India, less is known about how these interventions are implemented from the perspectives of the beneficiaries (patients and family caregivers) and healthcare professionals (HCPs).

India remains a very diverse country with vast differences in TB burden. The northern and central Indian states share more than half of the TB notifications, with relatively high TB prevalence as compared to other regions. In terms of socio-economic status, culture, nutritional status, and health-seeking behavior, which drives TB burden, there are stark differences at the state and regional levels, as reported in a recent nationwide survey of India [16,17]. Considering this complexity, the current qualitative study was conducted under National TB Elimination Program (NTEP) settings in various socio-cultural contexts and geographical locations across India, with the aim of gathering provider and beneficiary perspectives regarding nutritional support for persons with TB.

## 2. Methods

### 2.1. Study Design and Setting

Primary data from persons with TB undergoing treatment under India’s National TB Elimination Program, their family caregivers, and frontline HCPs, were included in this formative qualitative research study. This study was conducted in five states—Tamil Nadu, Karnataka, Maharashtra, Chhattisgarh, and Bihar—between August 2023 and April 2024. The southern, western, central, and northern states of India were represented by these states. India reported 1.5 million TB cases in the public sector in 2023, with Tamil Nadu reporting 64,690 (4%) cases, followed by Karnataka with 49,874 (3%) cases, Maharashtra with 110,059 (7%) cases, Chhattisgarh with 23,898 (1.5% cases), and Bihar with 76,461 (5%) cases. For this study, Madurai, a semi-urban district with 3931 TB cases; Kalaburagi, a semi-urban district with 2626 TB cases; Pune, an urban district with 2810 TB cases; Surguja, a tribal district with 2324 TB cases; and Patna, an urban district with 5202 cases in 2023, were chosen. From these study districts, both inpatient and outpatient clinics of the NTEP were chosen.

### 2.2. Study Population and Sampling

People with TB, who were receiving treatment under the NTEP in the study districts, their family caregivers, and medical staff made up the study population. We used criterion sampling to select persons representing different genders, age groups, and literacy levels. Socio-economic and cultural status was widely different between all the districts and was balanced across the sites. From the standpoint of treatment, the sample included individuals who received care in both inpatient and outpatient settings. The sample consisted of family caregivers nominated by persons with TB. The FGDs included frontline healthcare personnel, including nurses, health visitors, and senior treatment supervisors. Sixteen FGDs were held with TB patients, their caregivers, and frontline healthcare providers from several states.

### 2.3. Conceptual Framework and Data Collection Tools

We aimed to understand what was known, perceived, believed, and practiced in the context of nutrition and tuberculosis using the Knowledge, Attitude, and Practice (KAP) framework [18]. The KAP framework was utilized for assessing the behavioral and attitudinal factors which drove nutrition-related practices and the level of knowledge and perceptions underpinning such nutritional practices. The framework also provided insights into the diverse barriers, expectations, and needs in the context of nutrition and tuberculosis, especially in the service delivery aspect. Guided by the KAP framework, FGD guidelines were developed, adopted, and used across all participant categories (Table 1). Specifically, probes were made to understand the participants’ daily food intake routines and their understanding of the relationship between nutrition and TB. Experience in accessing nutrition-related information, nutritional support, and Direct Benefit Transfer provided by NTEP were inquired into. The kind of nutritional practices adopted during the treatment period were asked about, and expectations of the food and nutritional support expected from the TB program were investigated. Specifically, choices on nutritional and food delivery modes expected from the TB program were inquired about. Recommendations and strategies, with a focus on improving nutritional service delivery for TB patients, were also inquired about.

### 2.4. Participant Recruitment and Data Collection

Focus group discussions were used to collect primary data from three categories of participants—(i) adult persons with TB, (ii) family caregivers, and (iii) frontline healthcare providers—in all five districts under NTEP settings. Criterion sampling was used to recruit participants to ensure demographic representation. Participant selection happened at the public healthcare facilities under the NTEP of the study districts. Prior administrative permission was obtained from hospital officials, and the study staff explained the purpose of the research (with accompanying participant information) and invited the screened participants to take part. On expression of interest, all eligible interviewees were contacted and gathered at a time convenient to them for FGDs. Across all five districts, 81 adult persons with TB, 17 family caregivers, and 18 healthcare providers participated.

A primary list of open-ended questions, sub-questions, and related probes were prepared. The questions were structured in a way to generate maximum information in a systematic pattern from the respondents. All the FGDs were conducted in the local language, including Tamil, Kannada, Marathi, Chhattisgarh, and Hindi, by the field teams in each district. Each field team consisted of a team of data collectors and supervisors (M.M., N.R., S.K., B.U., N.R., S.D.B., M.P., N.K.). The data collection and supervision team consisted of male and female (N.R., S.D.B.) researchers who were trained in public health and social research and had an understanding of the local contexts. A site principal investigator for each district provided supportive supervision to ensure the quality of data collection and fidelity to the data collection guidelines. The FGD guidelines were revised and further FGDs were held in response to site-specific discussions. Patients and caregivers participated in separate FGDs. Depending on participant convenience, FGDs were held in both hospital and non-hospital settings. Each FGD lasted between 45 and 60 min, and they were recorded. The FGDs were led by a two-person field team responsible for note-taking and moderating. Confidentiality and privacy were maintained throughout the FGDs.

### 2.5. Data Analysis and Quality Control

The analysis team first transcribed and translated the recorded interviews verbatim into English, which they then read several times in order to become acquainted with the data narrative.

The quality of transcription and translation was evaluated by study investigators, considering linguistic nuances across the different states and cultures involved in the study. A preliminary analysis was conducted manually to understand the raw data and evolve the first lines of code. With an understanding of the emerging coding patterns, the FGD transcripts were anonymized and were imported into NVivo 12 (QSR International) software for thematic content analysis using an inductive approach.

Two authors (N.K. and Y.S.) independently examined the transcripts from every site in NVivo using the following procedures: (i) identifying a set of preliminary codes, (ii) identifying sub-themes, (iii) iteratively and consultatively defining and refining the sub-themes and themes, and (iv) generating the major themes. N.K. and Y.S.. conducted independent analyses to ensure reliability. Preliminary codes which reflected the granular-level awareness, knowledge, and experiences of the participants were exhaustively developed. Sub-themes were further evolved based on coherence and harmony between the preliminary codes. To ensure robustness, filed notes, memos, and patient feedback and views from the FGD moderators were used to enrich the coding process. The analysts matched the primary codes and themes to assess consistency. Disagreements were sorted out with input from site investigators. Data coding was continued until thematic data saturation was attained. The framework matrix method was used to compare the codes of the patients, family caregivers, and healthcare providers, and prominent quotes were selected. This approach provided an opportunity to triangulate and validate the mutual perspectives and experiences of patients, caregivers, and healthcare providers. Our reporting adheres to the Consolidated Criteria for Reporting Qualitative Research (COREQ) checklist, which was followed during this research [19]. The data collectors and analysts who were responsible for gathering and analyzing the data were trained in qualitative research and were fluent in English and local languages [Supplementary File S1].

### 2.6. Ethical Approval

The study was approved by the Central Ethics Committee of the Indian Council of Medical Research, New Delhi. The study details were explained to the participants and written consent was obtained from them. The study followed the National Ethical Guidelines for Biomedical and Health Research Involving Human Participants (2017, ICMR).

## 3. Results

In five districts, a total of 16 FGDs were conducted, with participation from 81 (79.8%) persons with TB, 17 (14.6%) family caregivers, and 18 (15.5%) healthcare providers. The majority of participants (84.4%) were literate, the mean age was 40, and 56.8% were male (Table 2).

### Coding Patterns

**Figure 1:**
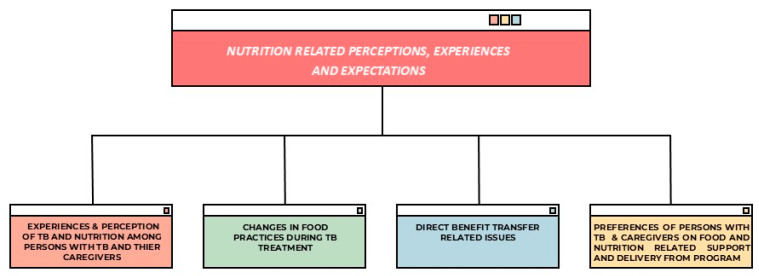
Themes related to nutrition-related perceptions, experiences, and preferences.

**Figure 2:**
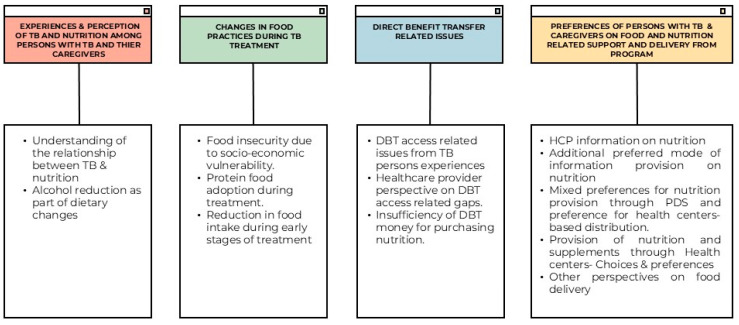
Sub-themes identified.

Twenty-eight preliminary codes were created from the transcripts of 16 FGDs. These codes were further classified into sub-themes and major themes. The themes were as follows: (a) the experiences and perceptions of TB and nutrition among persons with TB and their caregivers; (b) changes in food practices during TB treatment; (c) issues related to Direct Benefit Transfer; and (d) the preferences of TB patients and their caregivers regarding nutritional support and service delivery (Figure 1 and Figure 2).

**(A).**  Theme 1: Experiences and Perceptions of TB and Nutrition Among Persons with TB and Their Caregivers

Sub-Theme (i): Understanding of Relationship between TB and Nutrition

Participants pointed out that physical weakness or inadequate nutrition are risk factors for TB. Alcohol consumption, irregular food intake, missing meals, and a lack of a balanced diet were all frequently mentioned as risk factors or causes of tuberculosis. A person with TB (male, 25 years old) expressed the following:

*“Balanced nutrition is very important to gain the health; otherwise, the body system didn’t work and cause weakness. Due to lack of taking food on time, I am suffering this disease again after completing my first treatment”.*

Most persons with TB and their family caregivers had a clear understanding of the relationship between TB and malnutrition. Participants expressed that proper nutrition was essential for physical strength and wellness. The relationship between the body and food was presented as “a car which can only function properly if it has fuel in it”.

From the perspective of those with TB, proper food intake played an important role in overcoming challenges related to medication intake and treatment side effects. Participants highlighted that the quantity of food needs to be increased during the treatment period. A person affected by TB (female, 19 years old) expressed the following:

*“Less eating causes TB infection, Food should not be less. You should eat double the amount of food you eat.*

*“If eaten less food, you may become weak and then your immunity will be low”*

(Patient caregiver, 40 years Male, Pune)

Sub-Theme (ii): Alcohol Reduction as Part of Dietary Changes

Persons with tuberculosis highlighted that they had avoided drinking alcohol after the start of their treatment. A person with TB (male, 39 years old) who adhered to a treatment regimen consistently stated the following:

*“Before this, consumption of alcohol was there. After TB, only all kinds of healthy foods are taken by me sincerely”.*

Malnutrition was highlighted as a factor that contributed to the negative effects of alcohol. Healthcare providers also expressed that the government must restrict the sale of alcohol and tobacco, which could improve treatment compliance.

**(B).**  Theme 2: Changes in Food Practices During TB Treatment

Sub-Theme (i): Food Insecurity due to Socio-Economic Vulnerability

The prevalence of food insecurity was shared by a few participants from lower socio-economic statuses. Experiences of reducing or not being able to buy and consume nutrient-dense foods, particularly animal protein, were shared. In those circumstances, eggs and dal rice (pulses) were mentioned as popular substitutes. A person with TB (male, 26 years old, poor socio-economic status) expressed the following:

*“I am eating dal rice on daily basis before and after TB. As a rickshaw driver I don’t get enough money to buy any other supplements like chicken, mutton, fish in my diet. Because I’m financially weak, even sometimes due to lack of money I can arrange only one time food in a day”*

Healthcare providers also reported on the prevalent lack of nutritious food and how it affected patients based on socio-economic status. An HCP in Madurai expressed the following:

*“Cost of the food, spending for special food need is one of the major barrier to access balanced nutrition for all of them belonging to lower socio-economic status”*

Participants reported that the intake of protein-rich food was dependent on money availability and was inconsistent. A male patient, 35 years old, expressed the following:

*“I used to eat normal food like dal rice on daily basis. Sometimes I add eggs or milk in my diet before TB and same as after TB. Only sometimes I used to increase the little bit quantity of all this food depends upon the money which I get from my work”*

A male patient, 26 years old, from rural Patna expressed the following:

*“I don’t get the enough amounts to buy another supplements like chicken, mutton, fish in my diet. Because am financially weak. Even sometimes due to lack of money I can arrange only one time food in a day”*

Sub-Theme (ii): Protein Food Adoption During Treatment

Most persons with TB and family caregivers believed that having a diet high in protein was crucial for curing the disease. During the treatment period, a variety of protein-rich foods were reported to be consumed; individuals following a vegetarian diet tended to substitute paneer, soy, milk, and dal (pulses) for meat or eggs. Dry fruits, fruits, protein powder, moong dal (green gram beans), ragi (finger millet), urad dal (black/white gram), rice–wheat–millet porridge, and pongal (a boiled form of rice with ghee, pepper, and cumin) were other preferred food items during treatment. The intake of greens was considered important for overcoming the indigestion and acidity problems caused by medication intolerance. Ground nuts, almonds, millets, and chickpeas were also preferred by persons with TB. Green leaves were stressed as an important source of food and medicinal value by participants in Tamil Nadu. A family caregiver from Madurai, Tamil Nadu, opined the following:

*“Green leaf’s eg-murungaikeerai (moringa leaves) sirukeerai (tropical amarnath), pirandaithuvaiyal (adamant creeper-based dishes), thuthuvalai chutney and soup (solamum trilobatrum made dishes), manathakaalikeerai (black nigh shade)—give relief to stomach ulcers, araikeerai (spinach) helps in digestion. For protein—solam (sorghum), chickpeas (grams), nilakadalai (pea nut), badam (almond) will be helpful”.*

Some emphasized the value of eating protein-rich soups, such as crab soup, goat leg soup, cattle tail soup, and egg soup, which were believed to be helpful in relieving cough symptoms. Green leaves, potatoes, wheat, fluffy rice, bottle gourds, onions, garlic, lentils, milk, eggs, bananas, and occasionally fish were the nutrient-rich foods commonly mentioned by TB patients from tribal areas.

It was also emphasized that eating warm, freshly prepared food was crucial to recovery from tuberculosis. A person with TB, (female, 24 years old) expressed the following:

*“Mostly fresh food consumption now followed. Hot water consumption also followed due to symptoms. Now feeling better”.*

Sub-Theme (iii): Reduction in Food Intake During Early Stages of Treatment

While the importance of increased food intake and nutrition was the major theme of the responses, another aspect uncovered was the reduction in food intake during the early stages of treatment. It was noted that food intake had reduced, and issues of tastelessness, vomiting, and nausea were reported by participants. The intake of nutrition and food was noted to improve only gradually over the course of treatment after the initial months. A female family caregiver, 88 years old, opined as follows:

*“Nausea, vomiting, and wheezing interferes with adequate amount of food intake. Due to symptoms, it affect the food intake”*

**(C).**  Theme 3: Direct Benefit Transfer-Related Issues

In the context of nutritional support given by the TB program, the most often mentioned topic among TB patients and caregivers was the Direct Benefit Transfer (DBT) of INR 500, which is provided as a monthly payment for buying nutrition during treatment. Two topics were covered in the discussion.

Sub-Theme (i): DBT Access-Related Issues from Persons with TB’s Experiences

Persons with TB and their family caregivers frequently reported delays in receiving the DBT despite completing the necessary paperwork. One male caregiver, 40 years old, expressed the following:

*“We have completed and submitted every document which was required for the DBT money, but we have not got the helping money till date”.*

The lack of the timely provision of the DBT was underscored by a person with TB (male, 40 years old) from Patna, as follows:

*“Government should give the good amount of money at the time of treatment so that we can utilize it on that time of need for our diet and treatment. However, if we do not get the money in time, how can we have that protein rich diet? which is required at the time of treatment. And the money becomes meaningless if we are not getting it when we need it more.”*

Insufficient funds were often cited as a reason for delays in DBT provision by HCPs. It was also expressed, by persons with TB, that the DBT could be delivered through simpler methods (e.g., like money order) than at present, where it remains a cumbersome process.

Sub-theme (ii): Healthcare Provider Perspectives on DBT Access-Related Gaps

Healthcare providers listed a few important reasons regarding DBT access-related gaps: (i) a lack of a proper bank account for the beneficiaries; (ii) rural patients facing problems in opening zero-balance accounts at the post office; (iii) the lack of contact details of blood relations of patients to verify bank accounts; (iv) the lack of Aadhar card (Unique Identification Number) and bank account linkage; (v) the lack of verified addresses; and (vi) closed bank accounts due to a lack of income.

Sub-Theme (iii): Insufficiency of DBT Money for Purchasing Nutrition

The DBT amount was felt to be inadequate by most of the patients and their family caregivers. They indicated that the sum was insufficient to purchase the supplementary food, protein, and additional supplements necessary for the treatment. An enhancement of DBT support was frequently proposed as ideal. A person with TB (female, 18 years old) expressed the following:

*“We are facing many problems in the treatment due to lack of money because the medication charge, food charges and protein rich supplements charges are so high which we are not able to afford every time and this 500 per month will not cover the basic medications and nutritional needs”*

A female caregiver (40 years old) explained about the need for increasing DBT money:

*“Money can be increased, and if this cannot be done so at least all medicines should be provided from the center because buying medicines and other protein supplements are found costly from outside. Which common man can’t afford”*

**(D).**  Theme 4: Preferences of Persons with TB and Caregivers for Food- and Nutrition-Related Support and Delivery from Program

Sub-Theme (i): HCP Information on Nutrition

Persons with TB and family caregivers underscored that nutrition-related information was provided by the doctor and frontline HCPs at the treatment facilities. The HCPs gave crucial advice on the significance of consuming appropriate food (particularly protein-rich foods), when to eat, and how to take medications. A few participants agreed that people with diseases like diabetes require individualized nutritional counseling. A male person with TB said the following:

*“Yes! Doctors and DOTs provider of Patna center have told us about nutrition intake before or after intake of medicines. They also said about the protein rich diet like Paneer, soya beans for vegetarians and eggs, fish, chicken, mutton for non- vegetarians. They said about other nutritional items like protein powder, dry fruits etc. during the treatment initiation and follow-ups too”.*

Sub-Theme (ii): Additional Preferred Mode of Information Provision on Nutrition

Sharing food-related information through group discussion, counseling, and nutritional charts was preferred. A person with TB (female, 34 years old) opined as follows:

*“One day, I went earlier to the NTEP center and had time to go through all the charts pasted at the center. I had gone through them patiently. There were simplified food charts (what must be taken and not), with complete details about DBT and help line number was also provided there”.*

Information related to nutrition-related practices was expected by persons with TB. They also emphasized the importance of a telephonic helpline for nutrition-related assistance. In order to exchange nutritional experiences, healthcare providers insisted on nurturing patient support groups. Additionally, they emphasized the value of dietary charts and visual information. It was underscored that straightforward, affordable, and easily adaptable dietary recommendations would be more beneficial. Healthcare professionals stated that art performances and short films could be effective means of educating TB patients and their caregivers on a broad scale about nutrition.

Sub-Theme (iii): Mixed Preferences for Nutrition Provision through Public Distribution System (PDS) and Preference for Health-Center-Based Distribution

When given the hypothetical option of nutrition distribution, PDS stores were generally not chosen to supply food rations because of perceived leakage problems. A family caregiver (female, 60 years old) said the following:

*“The government is giving 5 kg food as a ration, but the distributing middleman reduces its quantity, and we get only 4 kg of the dry food item”.*

It was perceived by persons with TB that if the delivery of food rations was provided through PDS shops, there could be gaps in the timely communication from PDS shops to the beneficiaries. But some healthcare providers suggested the provision of prepared, packed, or ready-to-use foods through PDS shops. An HCP in Tamil Nadu opined as follows:

*“Supplementary foods enriched with essential nutrients (packed foods, health mix like preparations), if supplied under PDS, will be beneficial”.*

Sub-Theme (iv): Provision of Nutrition and Supplements through Health Centers—Choices and Preferences

The provision of food rations and supplements at health centers (where persons with TB take their medication) was the most-preferred delivery option for persons with TB and their caregivers. Dry food rations and packed food items were mostly preferred.

A family caregiver (female, 60 years old) expressed that “Medicines or nutritional supplements or dry food packets/ration should be provided to the center itself so that the patient can collect himself along with the medications without any mediator”.

Persons with TB expected to receive vitamin supplements and a liver tonic from health facilities, in addition to their prescription drugs, which they could not afford to purchase elsewhere. A few people and caregivers favored prepared foods, packed juices, and health mix preparations.

Healthcare providers from Tamil Nadu emphasized the benefits of providing F75 formula, protein meal, and millets at the health facility level. The HCPs also suggested providing millets from the health center. The to-door delivery of ration supplies was not desired by persons with TB due to concerns about leakage and reduction in food quantity. Rather, they insisted on collecting rations from the health centers while they came to collect medications. Participants did not prefer the option of mediators between themselves and the healthcare providers in the supply of rations.

Sub-Theme (v): Other Perspectives on Food Delivery

In addition to nutritional support, liver tonics and multi-vitamins in supplementation were expected from those in the TB program as they are very costly to procure outside of it. Food supplements, in addition to monetary support, were, in general, preferred by most of them. A person with TB (female, 19 years old) in Pune opined as follows:

*“Give money and give ration, as per how much ration is required for a patient. We also have to buy medicine”.*

Food rations, if provided, were requested for the whole family. The adoption of needy persons with TB by donors was expressed as a good solution for addressing nutritional needs. The following was expressed by a person with TB (male, 40 years old):

*“Government should give the ration for the whole family. As the person who is having the responsibility of the family only usually suffer from TB disease. He falls ill and not able to work and so how the family will survive. So, at least govt. should provide ration food for the whole family”.*

## 4. Discussion

This qualitative study of TB patients, caregivers, and healthcare providers across different socio-geographical regions of India offers insights into their knowledge, experiences, perceptions, and expectations about nutritional support and delivery. There was a proper understanding of the relationship between proper nutrition and TB cures among persons with TB and family caregivers [20,21]. Also, the role of nutrition in overcoming treatment challenges and side effects was widely recognized by them. Nutritional adaptation was found to be a common phenomenon among persons with TB, who have reportedly adopted some form of protein consumption, either from plant or animal-based foods [22]. Although an overall increase in food consumption was observed, participants also reported that their food intake decreased during the initial treatment phase due to difficulties including vomiting and nausea which impeded their weight-gain progress [23]. Thus, the provision of extranutritional support requires a gradual and individualized approach based on patients’ physical status.

Concerning the nutritional support offered by the TB program, individuals diagnosed with TB have predominantly reported difficulties in meeting their dietary needs with the DBT money. It was felt to be necessary to increase the DBT funding two-fold or thrice [24]. It was noted that DBT funds were often employed to acquire additional TB medication [25]. While treatment is provided fully free-of-cost for patients under the NTEP in India, patient experiences still show that they use DBT money to purchase additional supplements like vitamins, etc. DBT-related banking challenges, especially the delays in fund disbursement, significantly influence participants’ perceptions of governmental assistance for tuberculosis patients and required corrective actions from both providers and beneficiaries [25]. Money transfer by conventional transfer methods (postal method based on home address and in-person delivery) was preferred by some persons as simple and easy as compared to DBT [24]. Although basic nutrition-related information is already provided by healthcare providers, counseling, charts, visual aids, and short films were favored as ways of delivering comprehensive nutritional knowledge. Patient support groups were recommended as a modality to deliver nutrition-related information to patients. The implementation of diverse communication methods could achieve the dissemination of nutrition-related information in an efficient way.

Tailored nutritional advice for individuals with tuberculosis and co-morbidities (e.g., tuberculosis and diabetes) was requested, along with a preference for a telephonic helpline for nutritional information. While nutritional counseling by healthcare providers is being prioritized as an important intervention, the additional preferences identified in this study could be of importance for the TB program to consider [26].

New insights were noted concerning the expectations of individuals with tuberculosis and their caregivers regarding nutrition-related support from the government. When we inquired about possible nutritional delivery methods, a preference existed for the provision of food rations via NTEP health centers by medical workers. A reason for this preference could be due to the familiarity and communication which the patients have with their healthcare providers. However, there was a decreased preference for distribution via PDS outlets due to apprehensions of leakage. This may be attributed to the quality concerns related to service delivery via PDS outlets in India [27]. Also, the disclosure concerns associated with PDS-shop-based delivery to patients could be seen as an impediment for the patients. Participants were less preferential about the to-door delivery of food items and the involvement of middlemen in delivering food items. Here, again, disclosure concerns and unwillingness to engage with persons other than healthcare providers for nutrition delivery could be the underlying reason for this. The supplying of packed food items and protein mix through health centers was preferred. DBT money, together with food rations, was considered necessary by a few participants. There was a reduced preference for prepared meals, and healthcare personnel emphasized the adoption of patients by donors for nutritional support. There was a preference for supplying food for the entire family of persons with tuberculosis. A recent community-based trial conducted in India found that nutritional interventions for household contacts of TB patients resulted in a reduction in TB incidence among household members by 48%. Thus, the provision of nutritional support for household contacts could have an incremental impact on TB transmission reduction [2].

Overall, this study summarizes that persons with TB and family caregivers had a better understanding of the importance of nutrition in preventing and curing TB. The incorporation of protein consumption in various forms and the recognition of its significance were generally found among patients and caregivers. A lack of nutritious food and gaps in purchasing and consuming extra supplementary foods due to high costs were experienced. Socio-economic vulnerability was found, affecting nutritional choices and the consumption of food during treatment periods. While few reported a lack of protein-rich food, there were instances where patients living in poverty had to skip even routine food. A major issue which was underscored was that DBT provision was found to be insufficient and access to DBT was delayed due to bank-related issues. An increase in DBT amount by two-fold to three-fold was preferred by persons with TB and caregivers. In this instance, it is notable that, recently, the Government of India increased the DBT money two-fold for all patients under treatment.

Rations supply in the form of packed food items and supplementary nutrition through TB health centers was preferred. If offered, PDS-based ration distribution was less preferred and door delivery or the involvement of middlemen in ration distribution was the least preferred. DBT and nutritional supply in the form of packed food were recommended for better outcomes by healthcare providers. The availability of nutrition-related information through a telephonic helpline, visual information, and patient support-group-based discussion were mostly preferred. Our study had a few limitations. We used qualitative methods based on FGDs at one time point and may lack a temporal perspective and be subject to recall bias. To mitigate such issues and ensure the quality of our data, as per the consolidated criteria for reporting qualitative research, we took the following steps: (i) we engaged trained social researchers for conducting FGDs, (ii) established rapport with the participants in advance for better probing, and (iii) developed FGD probes and piloted them to ensure better sequencing and response elicitation. The direct observation and documentation of individual nutrition- and food-related practices and experiences were unfeasible, since this would require considerable time to be spent in the patients’ houses, which could lead to disclosure concerns. To mitigate this, we also included family caregivers’ insights into the food practices of the patient and triangulated their insights into our findings. The FGDs were conducted separately among patients and caregivers to ensure bias-free responses. Considering generalizability, this study was conducted among TB patients attending outpatient and inpatient clinics and among different ages, genders, locations and socio-economic statuses to obtain a comprehensive insight into nutrition-related preferences.

## 5. Conclusions

This study offers insights into nutritional support and delivery mechanisms for tuberculosis from the perspectives of both beneficiaries and suppliers. The subsequent recommendations are derived from this qualitative analysis. Protein intake, which was perceived by persons with TB and caregivers as the most important food requirement, could be increased in the nutritional package for persons with TB. Locally available plant and animal sources of protein could be considered as cost-effective options. The DBT provision may be considerably increased in accordance with socio-economic status and needs-based evaluations. Conducting socio-economic needs assessments among patients and providing financial support based on the incomes and unmet needs of the individuals could address nutritional inequity and ensure a continuum of the cascade of care [28]. Inter-sectoral collaboration with the banking sector to address bank account linkage and address verification could ensure the timely provision of the DBT for the patients. Establishing a dedicated call line or help center for DBT-related queries and concerns could alleviate and smoothen the process of DBT. The collection of socio-economic data could elucidate the needs of individuals and enhance the efficacy of interventions. An inter-sectoral approach between the TB program and the banking sector is needed to effectively address the DBT access-related issues in a timely manner. Concerns pertaining to Aadhar connection, mobile phone number discrepancies, address concerns, and having zero bank balance in relation to the DBT necessitate engagement and resolutions between patients, healthcare providers, and banks. Sensitizing and supporting the patients to complete DBT bank linkage-related tasks could be given importance. Public Distribution System (PDS)-based ration distribution, if implemented, must be tailored to the needs of TB program beneficiaries (disclosure concerns, timely delivery, reminder calls, etc.), and integration would require very close intersectoral coordination. The timely provision of food supplies through healthcare providers in health centers, with options for prepared, packed, and ready-to-use food, needs to be considered. Providing comprehensive nutritional information via a phone helpline, visual awareness tools, and patient support-group-based discussions could be implemented.

## Figures and Tables

**Table 1:** Focus group discussion probes.

Knowledge, awareness, and perception about causes of TB, treatment for TB, and prevention Knowledge, awareness, perception, and beliefs about risk factors for TB Knowledge, awareness, perception, and beliefs about balanced nutrition and malnutrition and their roles as protective and risk factors for TB Awareness and experiences of nutritional/food practices before and after TB which were adopted Perception about nutritional diet and its association with better adherence/treatment outcomes Gaps/barriers in terms of accessing nutritional/diet-related information and knowledge for patients Gaps/barriers in terms of preparing required nutritional diet for patients Gaps/barriers in terms of cost and accessibility in obtaining necessary dietary materials during treatment	Gaps/barriers in terms of affordability and accessibility in obtaining necessary dietary raw material products using Direct Benefit Transfer given by NTEP Expectations of healthcare providers in fulfilling nutritional needs of patients (information/counseling/diet charts, etc.) Expectation of NTEP in fulfilling nutritional needs of patients (DBT INR 500 experiences/food tokens/PDS ration shops/quantity of food/food types/raw or prepared/delivery mode, etc.) Expectation of NTEP program in fulfilling special nutritional needs of low-BMI/adolescent/diabetic/DT-TB patients (DBT/food tokens/quantity of food/food types/raw or prepared/delivery mode, etc.)

**Table 2:** Demographic characteristics of participants of FGDs.

Type of Participants	Total	Male No. (%)	Female No. (%)	Mean Age	Literate No. (%)	Illiterate No. (%)
Persons with TB	81	53 (65%)	28 (35%)	37	68 (84%)	14 (17%)
Family Caregivers	17	8 (47%)	9 (53%)	38	12 (71%)	5 (29%)
Healthcare Providers	18	5 (28%)	13 (72%)	46	18 (100%)	0 (0%)

## Data Availability

All relevant data are included in this publication. Audio files and transcripts of this study contain sensitive and personal information about patients and families and thus will not be shared to maintain participant confidentiality.

## Supplementary Materials

The following supporting information can be downloaded at: https://www.mdpi.com/article/10.3390/tropicalmed10040114/s1, File S1: COREQ checklist—Consolidated criteria for reporting qualitative studies (COREQ): 32-item checklist.
